# *S*,*S*′-Butane-1,4-diyl bis­(benzene­carbo­thio­ate)

**DOI:** 10.1107/S1600536813026822

**Published:** 2013-10-05

**Authors:** Daisuke Abe, Yuji Sasanuma

**Affiliations:** a Department of Applied Chemistry and Biotechnology, Chiba University, 1-33, Yayoi-cho, Inage-ku, Chiba 263-8522, Japan

## Abstract

The title compound, C_18_H_18_O_2_S_2_, which lies on an inversion center, adopts a *gauche* ^+^–*trans*–*trans*–*trans*–*gauche* ^−^ (*g* ^+^ *tttg* ^−^) conformation in the S—CH_2_—CH_2_—CH_2_—CH_2_—S bond sequence. In the crystal, mol­ecules are packed in a herringbone arrangement through inter­molecular C—H⋯π inter­actions.

## Related literature

For crystal structures and conformations of C_6_H_5_C(=O)S(CH_2_)_*n*_SC(=O)C_6_H_5_ (*n* = 2, 3, 5, 7, 9), see: for example, Deguire & Brisse (1988 ▶); Leblanc & Brisse (1992 ▶); Abe & Sasanuma (2012 ▶).
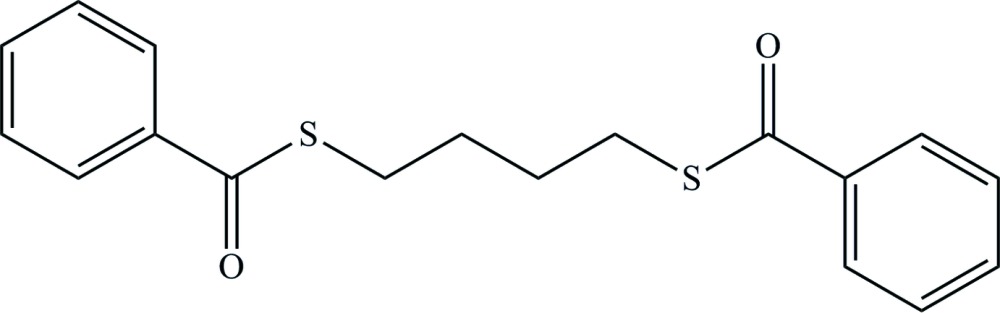


## Experimental

### 

#### Crystal data

C_18_H_18_O_2_S_2_
*M* *_r_* = 330.44
Monoclinic, 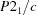

*a* = 13.2230 (14) Å
*b* = 4.8903 (5) Å
*c* = 13.2638 (15) Å
β = 106.897 (1)°
*V* = 820.67 (15) Å^3^
*Z* = 2
Mo *K*α radiation
μ = 0.33 mm^−1^
*T* = 173 K
0.30 × 0.30 × 0.05 mm

#### Data collection

Bruker APEXII CCD diffractometer
Absorption correction: multi-scan (*SADABS*; Bruker, 2001 ▶) *T*_min_ = 0.908, *T*_max_ = 0.984
4326 measured reflections
1845 independent reflections
1626 reflections with *I* > 2σ(*I*)
*R*_int_ = 0.015

#### Refinement

*R*[*F* ^2^ > 2σ(*F* ^2^)] = 0.030
*wR*(*F* ^2^) = 0.083
*S* = 1.05
1845 reflections
100 parameters
H-atom parameters constrained
Δρ_max_ = 0.21 e Å^−3^
Δρ_min_ = −0.24 e Å^−3^

### 

Data collection: *APEX2* (Bruker, 2007 ▶); cell refinement: *SAINT* (Bruker, 2007 ▶); data reduction: *SAINT*; program(s) used to solve structure: *SHELXS97* (Sheldrick, 2008 ▶); program(s) used to refine structure: *SHELXL97* (Sheldrick, 2008 ▶); molecular graphics: *SHELXTL* (Sheldrick, 2008 ▶) and *Mercury* (Macrae *et al.*, 2006 ▶); software used to prepare material for publication: *SHELXTL* and *PLATON* (Spek, 2009 ▶).

## Supplementary Material

Crystal structure: contains datablock(s) I. DOI: 10.1107/S1600536813026822/is5305sup1.cif

Structure factors: contains datablock(s) I. DOI: 10.1107/S1600536813026822/is5305Isup2.hkl

Additional supplementary materials:  crystallographic information; 3D view; checkCIF report

## Figures and Tables

**Table 1:** Hydrogen-bond geometry (Å, °) *Cg*1 is the centroid of the C1–C6 phenyl ring.

*D*—H⋯*A*	*D*—H	H⋯*A*	*D*⋯*A*	*D*—H⋯*A*
C4—H4⋯*Cg*1^i^	0.95	3.09	3.8810 (15)	141

Symmetry code: (i) 
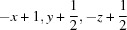
.

**Fig. 1.**
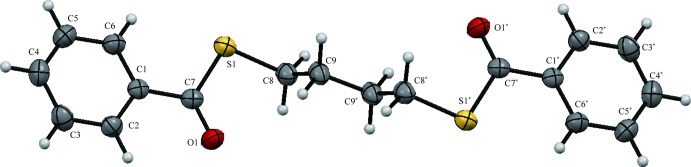
Molecular structure of S,S'-butane-1,4-diyl dibenzothioate (4DBS2). Displacement ellipsoids are drawn at the 50% probability level.

**Fig. 2.**
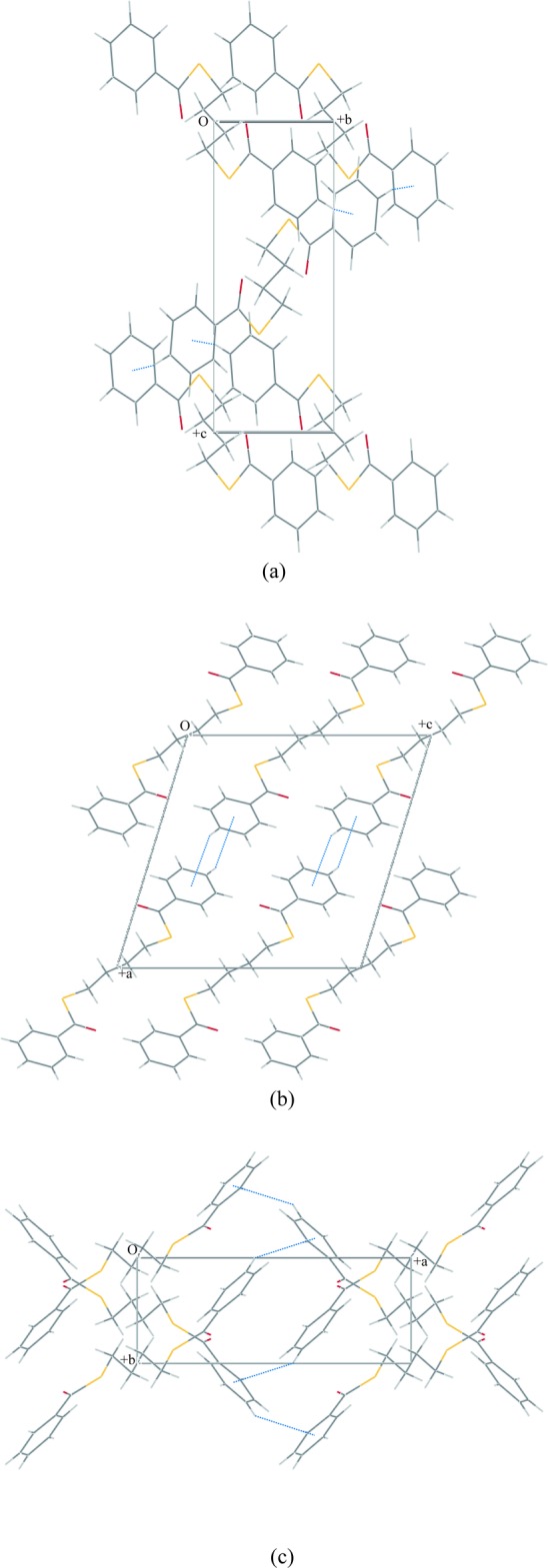
Packing diagram of 4DBS2, viewed down the (a) a, (b) b, and (c) c axes. The dotted lines represent C—H···π interactions.

## supplementary crystallographic information

### 1. Comment

In expectation of superior properties such as chemical and thermal resistance, we have investigated structures and properties of polythioesters ([–S(CH_2_)*_n_*SCOC_6_H_4_CO–]*_x_*, abbreviated as P*n*TS_2_), where *n* denotes the number of methylene units. Instead of the polymer itself, a small model compound corresponding to the repeating unit is often employed to elucidate conformational characteristics of the polymer; therefore, we have adopted oligomethylenedithiobenzoate (*n*DBS_2_). This paper describes synthesis and X-ray diffraction analysis of 4DBS_2_, a model compound of P4TS_2_.

The crystal structure of 2DBS_2_ was determined previously (Deguire & Brisse, 1988). In the 2DBS_2_ crystal, the S—CH_2_—CH_2_—S bonds lie in the *gauche*^+^ - *trans* - *gauche*^-^ (*g*^+^*tg*^-^) conformation. Our molecular orbital calculations and NMR experiments (Abe & Sasanuma, 2012) showed that this conformation is significantly stable even in isolated and liquid states owing to the anti-parallel arrangement of S—C═O dipole moments (the intramolecular dipole-dipole interaction).

Figure 1 shows the molecular structure of 4DBS_2_. The S—CH_2_—CH_2_—CH_2_—CH_2_—S part adopts the *g*^+^*tttg*^-^ conformation, and the intramolecular dipole-dipole interaction similar to that of 2DBS_2_ may be formed; however, 2DBS_2_ and 4DBS_2_ have markedly different melting points and densities: 94 °C and 1.41 g cm^-3^ (2DBS_2_); 49 °C and 1.34 g cm^-3^ (4DBS_2_). These differences may be partly due to strengths of intermolecular interactions. In the 2DBS_2_ crystal, a number of intermolecular interactions such as C═O···H—C, C—H···S, and C—H···π can be found. In contrast, the 4DBS_2_ crystal has only a few C—H···π interactions (Fig. 2).

Crystal structures of *n*DBS_2_ (*n* = 3, 5, 7, 9) were also reported (Leblanc & Brisse, 1992). The *n*DBS_2_ molecules adopt (*t*)*_n_ g*^+^ conformations in the S—(CH_2_)*_n_*—S part. Interestingly, the *n*DBS_2_ molecules show clear odd-even effects in the alkyl conformation: *g*^+^(*t*)*_n_*_-1_*g*^-^ (*n* = even); (*t*)*_n_**g*^+^ (*n* = odd).

### 2. Experimental

Benzoyl chloride (15.5 g, 0.11 mol) was added dropwise into 1,4-butanedithiol (6.1 g, 0.05 mol) and pyridine (8.7 g, 0.11 mol) kept at 0 °C, and then the mixture was stirred for 2 h. The crude product was diluted with diethyl ether (50 ml) and washed with water, 8% sodium hydrogen carbonate solution, and water. The organic layer was condensed, and white solid remained. The solid was recrystallized from ethanol to yield 4DBS_2_ (8.7 g, 53%).

The product was dissolved in chloroform in an open vessel. The vessel was placed in a larger one containing methanol, a poor solvent for 4DBS_2_, to facilitate precipitation of crystals by vapor diffusion of methanol into the chloroform solution.

### 3. Refinement

All C—H hydrogen atoms were geometrically positioned with C—H = 0.95 and 0.99 Å for the aromatic and methylene groups, respectively, and refined as riding with *U*_iso_(H) = 1.2*U*_eq_(C).

### Crystal data

**Table:**

C_18_H_18_O_2_S_2_	*F*(000) = 348
*M**_r_* = 330.44	*D*_x_ = 1.337 Mg m^−^^3^
Monoclinic, *P*2_1_/*c*	Melting point: 323 K
Hall symbol: -P 2ybc	Mo *K*α radiation, λ = 0.71073 Å
*a* = 13.2230 (14) Å	Cell parameters from 2016 reflections
*b* = 4.8903 (5) Å	θ = 3.2–26.8°
*c* = 13.2638 (15) Å	µ = 0.33 mm^−^^1^
β = 106.897 (1)°	*T* = 173 K
*V* = 820.67 (15) Å^3^	Plate, colourless
*Z* = 2	0.30 × 0.30 × 0.05 mm

### Data collection

**Table:**

Bruker APEXII CCD diffractometer	1845 independent reflections
Radiation source: fine-focus sealed tube	1626 reflections with *I* > 2σ(*I*)
Graphite monochromator	*R*_int_ = 0.015
Detector resolution: 8.333 pixels mm^-1^	θ_max_ = 27.5°, θ_min_ = 3.2°
φ and ω scans	*h* = −15→17
Absorption correction: multi-scan (*SADABS*; Bruker, 2001)	*k* = −6→6
*T*_min_ = 0.908, *T*_max_ = 0.984	*l* = −13→17
4326 measured reflections	

### Refinement

**Table:**

Refinement on *F*^2^	Primary atom site location: structure-invariant direct methods
Least-squares matrix: full	Secondary atom site location: difference Fourier map
*R*[*F*^2^ > 2σ(*F*^2^)] = 0.030	Hydrogen site location: inferred from neighbouring sites
*wR*(*F*^2^) = 0.083	H-atom parameters constrained
*S* = 1.05	*w* = 1/[σ^2^(*F*_o_^2^) + (0.0413*P*)^2^ + 0.1965*P*] where *P* = (*F*_o_^2^ + 2*F*_c_^2^)/3
1845 reflections	(Δ/σ)_max_ = 0.001
100 parameters	Δρ_max_ = 0.21 e Å^−^^3^
0 restraints	Δρ_min_ = −0.24 e Å^−^^3^

### Special details

**Table:**

Geometry. All e.s.d.'s (except the e.s.d. in the dihedral angle between two l.s. planes) are estimated using the full covariance matrix. The cell e.s.d.'s are taken into account individually in the estimation of e.s.d.'s in distances, angles and torsion angles; correlations between e.s.d.'s in cell parameters are only used when they are defined by crystal symmetry. An approximate (isotropic) treatment of cell e.s.d.'s is used for estimating e.s.d.'s involving l.s. planes.
Refinement. Refinement of *F*^2^ against ALL reflections. The weighted *R*-factor *wR* and goodness of fit *S* are based on *F*^2^, conventional *R*-factors *R* are based on *F*, with *F* set to zero for negative *F*^2^. The threshold expression of *F*^2^ > σ(*F*^2^) is used only for calculating *R*-factors(gt) *etc*. and is not relevant to the choice of reflections for refinement. *R*-factors based on *F*^2^ are statistically about twice as large as those based on *F*, and *R*-factors based on ALL data will be even larger.

### Fractional atomic coordinates and isotropic or equivalent isotropic displacement parameters (Å^2^)

**Table:**

	*x*	*y*	*z*	*U*_iso_*/*U*_eq_	
C1	0.70285 (9)	0.4894 (3)	0.15671 (9)	0.0292 (3)	
C2	0.62320 (10)	0.6555 (3)	0.09507 (10)	0.0350 (3)	
H2	0.6032	0.6402	0.0205	0.042*	
C3	0.57314 (11)	0.8429 (3)	0.14248 (12)	0.0422 (3)	
H3	0.5188	0.9562	0.1003	0.051*	
C4	0.60192 (12)	0.8656 (3)	0.25099 (12)	0.0414 (3)	
H4	0.5688	0.9982	0.2832	0.050*	
C5	0.67851 (12)	0.6964 (3)	0.31244 (11)	0.0427 (3)	
H5	0.6968	0.7092	0.3870	0.051*	
C6	0.72895 (11)	0.5077 (3)	0.26594 (10)	0.0376 (3)	
H6	0.7814	0.3905	0.3087	0.045*	
C7	0.75788 (10)	0.2993 (3)	0.10215 (10)	0.0311 (3)	
C8	0.91038 (11)	−0.0700 (3)	0.08957 (11)	0.0377 (3)	
H8A	0.9495	−0.2334	0.1244	0.045*	
H8B	0.8470	−0.1342	0.0344	0.045*	
C9	0.98021 (10)	0.0896 (3)	0.03740 (11)	0.0382 (3)	
H9A	1.0415	0.1647	0.0924	0.046*	
H9B	0.9397	0.2452	−0.0023	0.046*	
O1	0.73074 (8)	0.2677 (2)	0.00745 (7)	0.0429 (3)	
S1	0.86863 (3)	0.12549 (8)	0.18584 (3)	0.03886 (13)	

### Atomic displacement parameters (Å^2^)

**Table:**

	*U*^11^	*U*^22^	*U*^33^	*U*^12^	*U*^13^	*U*^23^
C1	0.0297 (6)	0.0284 (6)	0.0294 (6)	−0.0041 (5)	0.0084 (5)	0.0010 (5)
C2	0.0353 (7)	0.0401 (7)	0.0308 (6)	0.0017 (5)	0.0115 (5)	0.0074 (5)
C3	0.0405 (7)	0.0437 (8)	0.0455 (8)	0.0097 (6)	0.0174 (6)	0.0129 (6)
C4	0.0428 (8)	0.0397 (7)	0.0477 (8)	0.0029 (6)	0.0224 (6)	−0.0016 (6)
C5	0.0469 (8)	0.0496 (9)	0.0322 (7)	0.0018 (7)	0.0123 (6)	−0.0050 (6)
C6	0.0392 (7)	0.0415 (8)	0.0294 (6)	0.0040 (6)	0.0057 (5)	0.0006 (6)
C7	0.0315 (6)	0.0308 (6)	0.0295 (6)	−0.0024 (5)	0.0063 (5)	0.0014 (5)
C8	0.0353 (7)	0.0341 (7)	0.0414 (7)	0.0042 (5)	0.0076 (6)	−0.0029 (6)
C9	0.0319 (7)	0.0347 (7)	0.0471 (8)	0.0018 (5)	0.0099 (6)	−0.0062 (6)
O1	0.0479 (6)	0.0495 (6)	0.0282 (5)	0.0103 (5)	0.0060 (4)	−0.0027 (4)
S1	0.0346 (2)	0.0456 (2)	0.0328 (2)	0.00686 (14)	0.00426 (14)	−0.00058 (14)

### Geometric parameters (Å, º)

**Table:**

C1—C6	1.3913 (17)	C6—H6	0.9500
C1—C2	1.3916 (17)	C7—O1	1.2117 (15)
C1—C7	1.4912 (17)	C7—S1	1.7761 (13)
C2—C3	1.3841 (19)	C8—C9	1.5204 (19)
C2—H2	0.9500	C8—S1	1.8053 (14)
C3—C4	1.382 (2)	C8—H8A	0.9900
C3—H3	0.9500	C8—H8B	0.9900
C4—C5	1.377 (2)	C9—C9^i^	1.526 (3)
C4—H4	0.9500	C9—H9A	0.9900
C5—C6	1.384 (2)	C9—H9B	0.9900
C5—H5	0.9500		
			
C6—C1—C2	119.39 (12)	C1—C6—H6	120.0
C6—C1—C7	122.48 (11)	O1—C7—C1	122.90 (12)
C2—C1—C7	118.13 (11)	O1—C7—S1	121.94 (10)
C3—C2—C1	119.98 (12)	C1—C7—S1	115.15 (9)
C3—C2—H2	120.0	C9—C8—S1	113.65 (10)
C1—C2—H2	120.0	C9—C8—H8A	108.8
C4—C3—C2	120.21 (13)	S1—C8—H8A	108.8
C4—C3—H3	119.9	C9—C8—H8B	108.8
C2—C3—H3	119.9	S1—C8—H8B	108.8
C5—C4—C3	120.04 (13)	H8A—C8—H8B	107.7
C5—C4—H4	120.0	C8—C9—C9^i^	111.70 (14)
C3—C4—H4	120.0	C8—C9—H9A	109.3
C4—C5—C6	120.26 (13)	C9^i^—C9—H9A	109.3
C4—C5—H5	119.9	C8—C9—H9B	109.3
C6—C5—H5	119.9	C9^i^—C9—H9B	109.3
C5—C6—C1	120.06 (13)	H9A—C9—H9B	107.9
C5—C6—H6	120.0	C7—S1—C8	100.22 (6)
			
C6—C1—C2—C3	2.1 (2)	C6—C1—C7—O1	174.55 (14)
C7—C1—C2—C3	−177.31 (13)	C2—C1—C7—O1	−6.1 (2)
C1—C2—C3—C4	−0.1 (2)	C6—C1—C7—S1	−6.66 (18)
C2—C3—C4—C5	−1.8 (2)	C2—C1—C7—S1	172.72 (11)
C3—C4—C5—C6	1.7 (2)	S1—C8—C9—C9^i^	175.95 (10)
C4—C5—C6—C1	0.4 (2)	O1—C7—S1—C8	−0.26 (14)
C2—C1—C6—C5	−2.3 (2)	C1—C7—S1—C8	−179.07 (11)
C7—C1—C6—C5	177.12 (14)	C9—C8—S1—C7	83.52 (11)

Symmetry code: (i) −*x*+2, −*y*, −*z*.

### Hydrogen-bond geometry (Å, º)

*Cg*1 is the centroid of the C1–C6 phenyl ring.

**Table:**

*D*—H···*A*	*D*—H	H···*A*	*D*···*A*	*D*—H···*A*
C4—H4···*Cg*1^ii^	0.95	3.09	3.8810 (15)	141

Symmetry code: (ii) −*x*+1, *y*+1/2, −*z*+1/2.
